# Supplementary material for: From sequence to enzyme mechanism using multi-label machine learning
Source: BMC Bioinformatics. 2014 May 19;15:150. doi: 10.1186/1471-2105-15-150 (PMC4229970; doi:10.1186/1471-2105-15-150)
Supplement: Additional file 2 — Java code of ml2db. Additional file ml2db_code.tar.gz contains the Java source code to run the multi-label machine learning experiments and save the results to database. The code’s Javadoc is included. [file 1471-2105-15-150-S2.zip › additional file 2/ml2db/utils/doc/uk/ac/standrews/utils/main/database/TableReader.html]

TableReader


JavaScript is disabled on your browser.


- Overview
- Package
- Class
- Use
- Tree
- Deprecated
- Index
- Help

- Prev Class
- Next Class

- Frames
- No Frames

- All Classes

- Summary:
- Nested |
- Field |
- Constr |
- Method

- Detail:
- Field |
- Constr |
- Method


uk.ac.standrews.utils.main.database

## Class TableReader

- java.lang.Object
- - uk.ac.standrews.utils.main.database.Manager
  - - uk.ac.standrews.utils.main.database.DbManager
    - - uk.ac.standrews.utils.main.database.TableManager
      - - uk.ac.standrews.utils.main.database.TableReader

- All Implemented Interfaces:
  :   java.io.Serializable

  ---

    

  ```
  public class TableReader
  extends TableManager
  ```

  Class

  Version:
  :   10 Jun 2010

  Author:
  :   Luna De Ferrari luna.deferrari-at-ed.ac.uk

  See Also:
  :   Serialized Form

- - ### Field Summary

    - ### Fields inherited from class uk.ac.standrews.utils.main.database.DbManager

      `m_dbWriter`
  - ### Constructor Summary

    Constructors

    Constructor and Description

    `TableReader(Table table)`
  - ### Method Summary

    Methods

    | Modifier and Type | Method and Description |
    | --- | --- |
    | `boolean` | `columnExistsInTable(java.lang.String column)` |
    | `TableRow` | `getRowValues(java.sql.ResultSet resultSet, int rowNumber)` Get the nth row of a `ResultSet` |
    | `java.util.Vector<java.lang.String>` | `getTableColumnsNames()` Gets the list of columns for a database table (from: http://forums.sun.com/thread.jspa?threadID=501222&messageID=2369725) |
    | `java.util.Vector<TableRow>` | `getTableRowsWhereEqual(java.lang.String column, java.lang.String value)` Gets the table rows where the given column has exactly the given value |
    | `TableRow` | `getTableRowWhereEqual(java.lang.String column, java.lang.String value)` Gets the \*first\* table row where the given column has exactly the given value |
    | `TableRow` | `getTableRowWhereLike(java.lang.String column, java.lang.String value)` Gets the \*first\* table row where the given column has a value similar to the given value |
    | `boolean` | `isEmpty(java.sql.ResultSet resultSet)` Returns true if the resultset is null or empty. |
    | `boolean` | `valueExistsInTable(java.lang.String column, java.lang.String value)` Checks whether a certain row value exists in the given table. |
    | `boolean` | `valuesExistsInTable(java.lang.String column1, java.lang.String value1, java.lang.String column2, java.lang.String value2)` Checks whether a row with the two given values exists in the given table. |
    | `boolean` | `valuesExistsInTable(java.lang.String column1, java.lang.String value1, java.lang.String column2, java.lang.String value2, java.lang.String column3, java.lang.String value3)` |

    - ### Methods inherited from class uk.ac.standrews.utils.main.database.TableManager

      `getTable, getTableName`
    - ### Methods inherited from class uk.ac.standrews.utils.main.database.DbManager

      `closeConnection, connectionIsValid, getDatabaseName, getDbConnection, getDbCreator, getDbReader, getDbWriter, refreshConnection, runAnalyzeTable`
    - ### Methods inherited from class java.lang.Object

      `equals, getClass, hashCode, notify, notifyAll, toString, wait, wait, wait`

- - ### Constructor Detail


    - #### TableReader

      ```
      public TableReader(Table table)
      ```
  - ### Method Detail


    - #### columnExistsInTable

      ```
      public boolean columnExistsInTable(java.lang.String column)
      ```


    - #### getRowValues

      ```
      public TableRow getRowValues(java.sql.ResultSet resultSet,
                          int rowNumber)
      ```

      Get the nth row of a `ResultSet`

      Parameters:
      :   `resultSet` -
      :   `rowNumber` - (starting at 0)

      Returns:
      :   the row


    - #### getTableColumnsNames

      ```
      public java.util.Vector<java.lang.String> getTableColumnsNames()
      ```

      Gets the list of columns for a database table (from:
      http://forums.sun.com/thread.jspa?threadID=501222&messageID=2369725)

      Returns:
      :   a list of table columns


    - #### getTableRowWhereEqual

      ```
      public TableRow getTableRowWhereEqual(java.lang.String column,
                                   java.lang.String value)
      ```

      Gets the \*first\* table row where the given column has exactly the given
      value

      Parameters:
      :   `column` - a table column name
      :   `value` - a value to be found in that column

      Returns:
      :   the \*first\* table row where the given column has exactly the
          given value


    - #### getTableRowsWhereEqual

      ```
      public java.util.Vector<TableRow> getTableRowsWhereEqual(java.lang.String column,
                                                      java.lang.String value)
      ```

      Gets the table rows where the given column has exactly the given value

      Parameters:
      :   `column` - a table column name
      :   `value` - a value to be found in that column

      Returns:
      :   the table rows where the given column has exactly the given value


    - #### getTableRowWhereLike

      ```
      public TableRow getTableRowWhereLike(java.lang.String column,
                                  java.lang.String value)
      ```

      Gets the \*first\* table row where the given column has a value similar to
      the given value

      Parameters:
      :   `column` - a table column name
      :   `value` - a value to be found in that column

      Returns:
      :   the \*first\* table row where the given column has a value similar
          to the given value


    - #### isEmpty

      ```
      public boolean isEmpty(java.sql.ResultSet resultSet)
      ```

      Returns true if the resultset is null or empty. Emptyness checked using
      the method: if resultSet.isBeforeFirst() is false, then the resultset
      must be empty http://forums.sun.com/thread.jspa?threadID=481745

      Parameters:
      :   `resultSet` -

      Returns:
      :   true if the resultset is null or empty


    - #### valueExistsInTable

      ```
      public boolean valueExistsInTable(java.lang.String column,
                               java.lang.String value)
      ```

      Checks whether a certain row value exists in the given table.

      Parameters:
      :   `value` - the row value
      :   `column` - the table column

      Returns:
      :   true if a row exists with that column value


    - #### valuesExistsInTable

      ```
      public boolean valuesExistsInTable(java.lang.String column1,
                                java.lang.String value1,
                                java.lang.String column2,
                                java.lang.String value2)
      ```

      Checks whether a row with the two given values exists in the given table.

      Parameters:
      :   `value` - the row value
      :   `column` - the table column

      Returns:
      :   true if a row exists with that column value


    - #### valuesExistsInTable

      ```
      public boolean valuesExistsInTable(java.lang.String column1,
                                java.lang.String value1,
                                java.lang.String column2,
                                java.lang.String value2,
                                java.lang.String column3,
                                java.lang.String value3)
      ```


- Overview
- Package
- Class
- Use
- Tree
- Deprecated
- Index
- Help

- Prev Class
- Next Class

- Frames
- No Frames

- All Classes

- Summary:
- Nested |
- Field |
- Constr |
- Method

- Detail:
- Field |
- Constr |
- Method
